# Generation of H7N9-specific human polyclonal antibodies from a transchromosomic goat (caprine) system

**DOI:** 10.1038/s41598-018-36961-5

**Published:** 2019-01-23

**Authors:** Hua Wu, Zhiqiang Fan, Michelle Brandsrud, Qinggang Meng, Molly Bobbitt, Misha Regouski, Rusty Stott, Alexis Sweat, Jackelyn Crabtree, Robert J. Hogan, Ralph A. Tripp, Zhongde Wang, Irina A. Polejaeva, Eddie J. Sullivan

**Affiliations:** 1 SAB Biotherapeutics, Sioux Falls, SD 57104 USA; 2 SAB Capra, LLC, Salt Lake City, UT 84101 USA; 3 Department of Animal, Dairy, and Veterinary Sciences, Utah State University, Logan, UT 84322 USA; 4 Department of Infectious Diseases, College of Veterinary Medicine, University of Georgia, Athens, GA 30602 USA

## Abstract

To address the unmet needs for human polyclonal antibodies both as therapeutics and diagnostic reagents, building upon our previously established transchromosomic (Tc) cattle platform, we report herein the development of a Tc goat system expressing human polyclonal antibodies in their sera. In the Tc goat system, a human artificial chromosome (HAC) comprising the entire human immunoglobulin (Ig) gene repertoire in the germline configuration was introduced into the genetic makeup of the domestic goat. We achieved this by transferring the HAC into goat fetal fibroblast cells followed by somatic cell nuclear transfer for Tc goat production. Gene and protein expression analyses in the peripheral blood mononuclear cells (PBMC) and the sera, respectively, of Tc caprine demonstrated the successful expression of human Ig genes and antibodies. Furthermore, immunization of Tc caprine with inactivated influenza A (H7N9) viruses followed by H7N9 Hemagglutinin 1 (HA1) boosting elicited human antibodies with high neutralizing activities against H7N9 viruses *in vitro*. As a small ungulate, Tc caprine offers the advantages of low cost and quick establishment of herds, therefore complementing the Tc cattle platform in responses to a range of medical needs and diagnostic applications where small volumes of human antibody products are needed.

## Introduction

Human polyclonal antibodies, also called intravenous immunoglobulins (IVIGs) or simply as immunoglobulin G (IgG), prepared from plasma of the general population or convalescing human donors have been used as therapeutic agents for treating a variety of diseases, such as immunodeficiency, infection, and autoimmunity^1^. However, because of the nature of voluntary donation of human plasma, the production of human IVIG therapeutics is limited by the shortage of supplies; there are also great concerns over the potential transmission of human diseases from donors to patients. Animal-derived polyclonal antibodies have also been used as therapeutics for certain disease indications, one of which being the life-saving anti-venoms derived from horse sera^2^. These animal-derived polyclonal antibody products also suffer from some significant limitations, chief among which are the severe adverse effects such as allergic reactions caused by the high immunogenicity of these products in humans^2^.

To address the limitation of human- and animal-derived polyclonal antibodies, we at SAB Biotherapeutics, Inc. have developed a transchromosomic (Tc) bovine platform with the capability to produce large quantities of highly potent fully-human polyclonal antibodies^3,4^. In the Tc bovine, the bovine immunoglobulin genes were genetically inactivated by gene targeting and the Ig functions were reconstituted by a human artificial chromosome (HAC) comprising the entire unrearranged human immunoglobulin heavy-chain (h*IGH*), kappa-chain (h*IGK*) and lambda-chain (h*IGL*) germline loci. Consequently, Tc cattle express fully-human IgG (hIgG)^5,6^. Through further optimizing the genetic elements on the HAC that are involved in B cell signaling and Ig class switch recombination, physiological levels of hIgG are produced in the blood of Tc bovine (2–9 mg/ml), leading to the production of 300–600 g of hIgG in a Tc bovine per month^6^. Equally important, these Tc cattle can be hyperimmunized with a pathogen of choice to produce highly potent pathogen-specific hIgG which have been successfully used to treat a list of viral and bacterial infection diseases in animal models, and some of these hIgG products have entered into human clinical trials^7–15^.

Building upon this Tc bovine platform, we recently initiated efforts to establish a Tc goat (*Capra aegagrus hircus*) system to produce hIgG in smaller ungulates. Development of a Tc system in a small ungulate species such as the goat is better suited for producing smaller volumes of hIgG for certain applications, including using hIgG as diagnostic reagents for serological testing of emerging diseases and as therapeutics for spotty outbreaks of infectious diseases and/or personalized therapeutic hIgG products targeting specific diseases. Goats also offer the advantages of having a shorter gestation period and growing to adult size much faster than larger ungulates, decreasing the time for herd development at much lower costs. Furthermore, because large numbers of Tc goats can be quickly produced, plasma from sufficient numbers of animals can be included in plasma pooling strategies to ensure a greater lot to lot consistency.

As the first step to establish the Tc goat system, we investigated whether the HAC we engineered is functional in the goat by supporting the expression of hIgG in Tc goat sera. Towards this goal, we transferred HACs into wildtype goat fetal fibroblast cells followed by somatic cell nuclear transfer (SCNT) to clone Tc goats. Here, we report that the produced Tc caprine expresses hIgG in the PBMCs and sera. We also further demonstrated that, with a H7N9 influenza A immunization study, the Tc caprine can be hyperimmunized and respond well to a pathogen of choice to produce pathogen-specific hIgG in the serum. Furthermore, functional studies on the elicited hIgG demonstrated high neutralizing activities against H7N9 viruses *in vitro*.

## Results

### Generation of Tc goat

Previously, we engineered a HAC, named isKcHAC∆, that comprises the entire human Ig genetic repertoire in the germline configuration in which the regulatory genomic sequences involved in pre-B cell receptor (preBCR) and BCR signaling during B cell development and those mediating human Ig class switch recombination are replaced with the respective genomic sequences from an ungulate (bovine)^6^. We have demonstrated in the Tc bovine system that the ungulate regulatory sequences engineered into the HAC allows high levels of hIgG expression and a hIgG subclass distribution with IgG1 dominancy, closely mimicking hIgG expression in humans^6^. We reasoned that these ungulate regulatory elements, due to their high similarity in sequence among ungulates, may also render the HAC to function well in Tc goats. Based on this rationale, we established single cell-derived Tc goat fibroblast cell colonies by transferring the isKcHAC∆ into goat fetal fibroblast cells via microcell-mediated chromosome transfer (MMCT) and used the produced Tc goat fibroblasts as nuclear donors for Tc goat production by SCNT. In total, 194 cloned embryos were generated using five isKcHAC∆ containing fetal fibroblast colonies and were transferred into 14 estrus-synchronized does (Table 1).

**Table 1:** *In vivo* development of embryos cloned from isKcHAC∆ containing fetal fibroblast colonies

Somatic cell donor colonies	No. of embryos transferred	No. of recipients	No. of pregnancies (% of recipients)			No. of live offspring (% of recipients)
			Day 40	Day 60	Day 90	
#4	22	2	0 (0)	0 (0)	0 (0)	0 (0)
#10	60	5	2 (40)	1 (20)	1 (20)	1 (20)
#13	46	3	0 (0)	0 (0)	0 (0)	0 (0)
#18	33	2	2 (100)	1 (50)	1* (50)	0 (0)
#35	33	2	0 (0)	0 (0)	0 (0)	0 (0)
**Total**	**194**	**14**	**4 (28.6)**	**2 (14.3)**	**2 (14.3)**	**1 (7.1)**

^*^This pregnancy was lost at day 129 of gestation.

**Figure 1:**
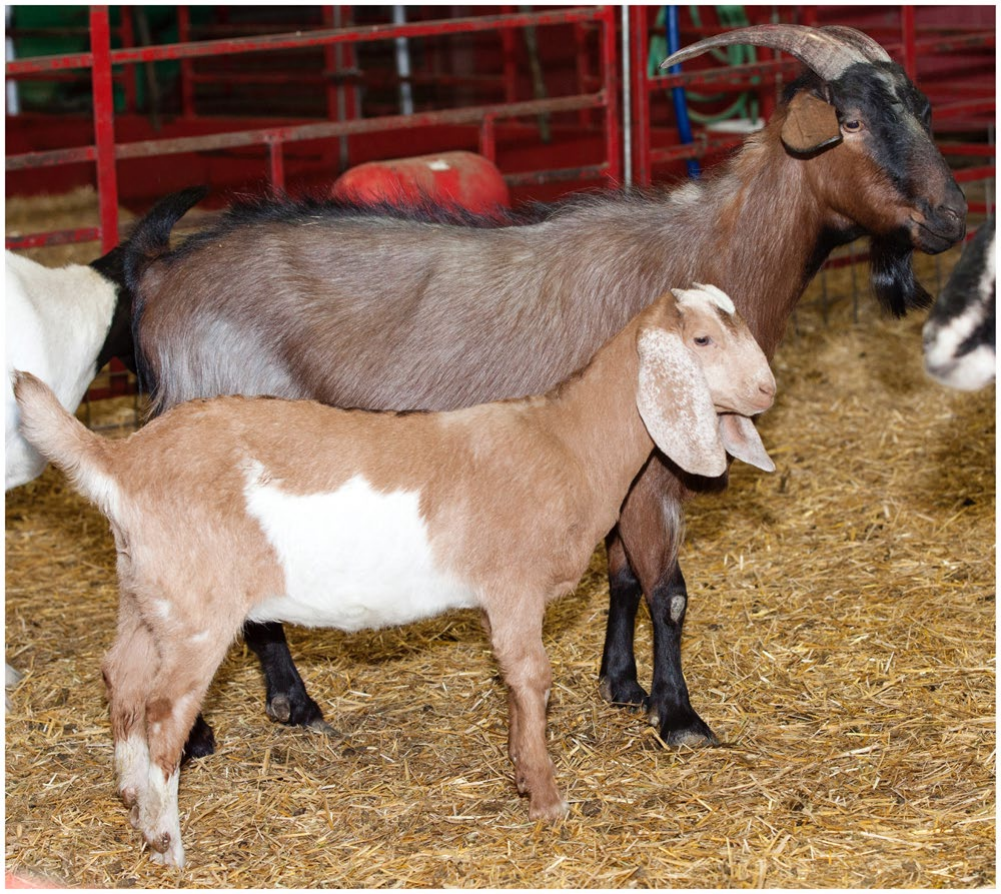
A transchromosomic (Tc) goat at 2 months of age next to her surrogate mother.

Pregnancy checks conducted with ultrasonography at day 40±3 of gestation confirmed that 4 out of 14 (28.6%) recipients were pregnant (Table 1). The pregnancies were obtained only from colonies #10 and #18. Two of the pregnancies aborted between day 40 and day 60 of gestation, and one more had a mummified fetus that was lost at day 129 of gestation. One pregnancy initiated from colony #10 went to term and resulted in the birth of a healthy Tc goat (Fig. 1).

### Genetic and serological characterization of the Tc goat

**Figure 2:**
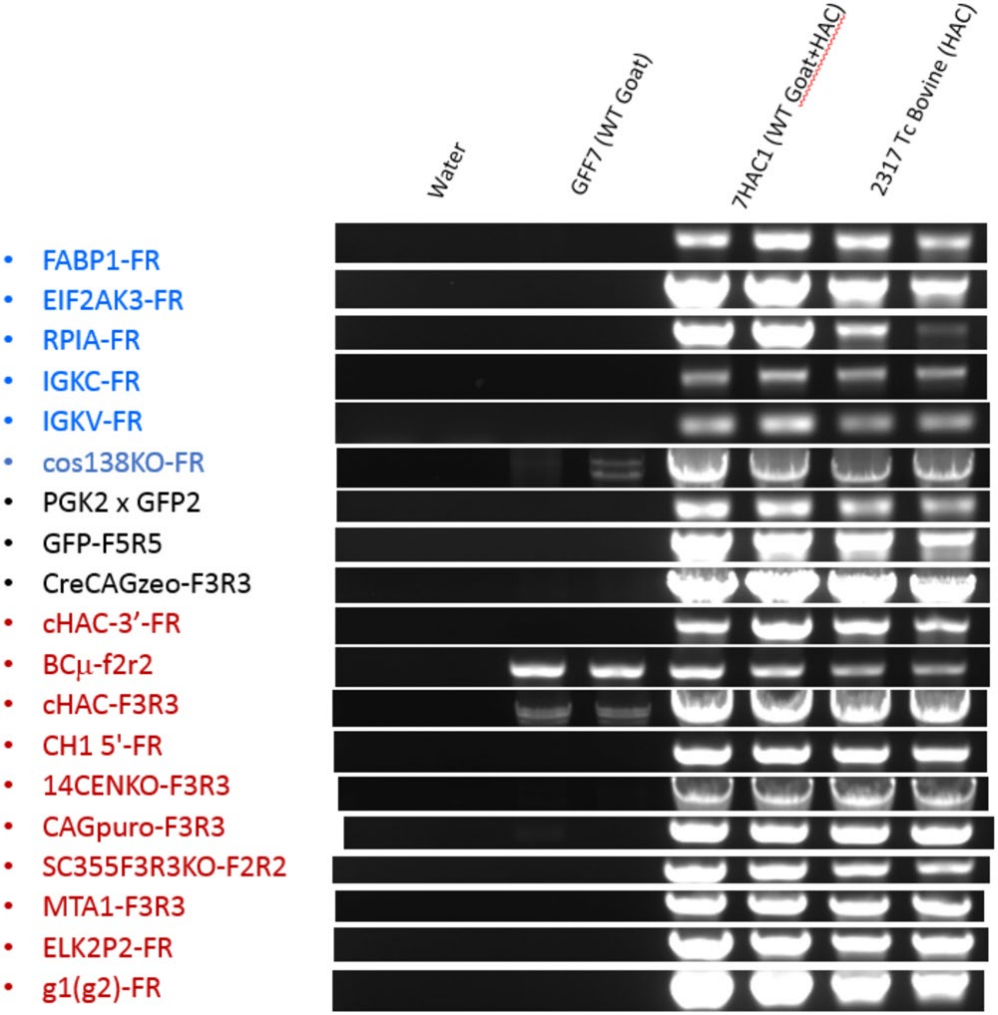
Genomic PCR on PBMC from Tc goat. PCR products for some of the junction points examined in the isKcHACΔ by using genomic DNA isolated from PBMCs of the Tc goat (7HAC1). Tc bovine 2317 containing isKcHACΔ was used as the positive control and wild type goat fetal fibroblasts (GFF7) before HAC introduction were as the negative control. This gel image was constructed from multiple original gel images as shown in the Supplementary Information file.

To confirm that the HAC has been retained in the Tc goat, we conducted genomic PCR analysis on peripheral blood mononuclear cells (PBMC) isolated from the Tc goat at birth with PCR primers specific to different domains of the HAC (Table 2). As shown in Fig. 2, all the PCRs performed were positive confirming the presence and structural integrality of the HAC in the Tc goat. We also subjected the PCR products to Sanger sequencing to validate the specificity of PCR.

**Table 2:** Sequences of PCR primers used for analyzing HAC retention and integrity in Tc goat blood cells.

Primer Name	Sequence
FABP1-F	5′-TATCAAGGGGGTGTCGGAAATCGTG-3′
FABP1-R	5′-ACTGGGCCTGGGAGAACCTGAGACT-3′
EIF2AK3-F	5′-AGGTGCTGCTGGGTGGTCAAGT-3'
EIF2AK3-R	5′-GCTCCTGCAAATGTCTCCTGTCA-3′
RPIA-F	5′-CTTACCCAGGCTCCAGGCTCTATT-3'
RPIA-R	5′-CTCTACCTCCCTACCCCATCATCAC-3′
IGKC-F	5′-TGGAAGGTGGATAACGCCCT-3'
IGKC-R	5′-TGGAAGGTGGATAACGCCCT-3′
IGKV-F	5′-AGTCAGGGCATTAGCAGTGC-3'
IGKV-R	5′-GCTGCTGATGGTGAGAGTGA-3′
PGK2	5′-TGTTCTCCTCTTCCTCATCTCC-3′
GFP2	5′-TGAAGGTAGTGACCAGTGTTGG-3′
GFP-F5	5′-TGGAACTGGATGGCGATGTGAATGG-3′
GFP-R5	5′-GGTAATGGTTGTCTGGGAGGAGCAC-3′
CreCAGzeo-F3	5′-GCCCTCACCTTGCAGACCACCTCCATCAT-3'
CreCAGzeo-R3	5′-CCTCTCCTGCTCAGTCCCCTTCCTTCCATC-3'
CH1 5′-F	5′-CCGACAGGCAGGGCACGAGGAG-3'
CH1 5′-R	5′-TGCGAGGCGGGACAAAGACAC-3′
14CENKO-F3	5′-ACTGAAATATTTTAAATGTTTGCCCTTCCCACTCC-3'
14CENKO-R3	5′-AGACCTCCGCGCCCCGCAACCTCCCCTTCTAC-3'
CAGpuro-F3	5′-GCGGCGCCGGCAGGAAGGAAATG-3'
CAGpuro-R3	5′-CGAGGCGCACCGTGGGCTTGTA-3'
SC355F3R3KO-F2	5′-GCCATTGTCGAGCAGGTAGT-3'
SC355F3R3KO-R2	5′-TCCCTCATCAGCCATCCTAA-3′
MTA1-F3	5′-AGCACTTTACGCATCCCAGCATGT-3′
MTA1-R3	5′-CCAAGAGAGTAGTCGTGCCCCTCA-3′
ELK2P2-F	5′-CCCACTTTACCGTGCTCATT-3′
ELK2P2-R	5′-ATGAAGGTCCGTGACTTTGG-3′
g1(g2)-F	5′-ACCCCAAAGGCCAAACTCTCCACTC-3′
g1(g2)-R	5′-CACTTGTACTCCTTGCCATTCAGC-3′
VH3-F	5′-AGTGAGATAAGCAGTGGATG-3′
VH3-R	5′-CTTGTGCTACTCCCATCACT-3′

**Figure 3:**
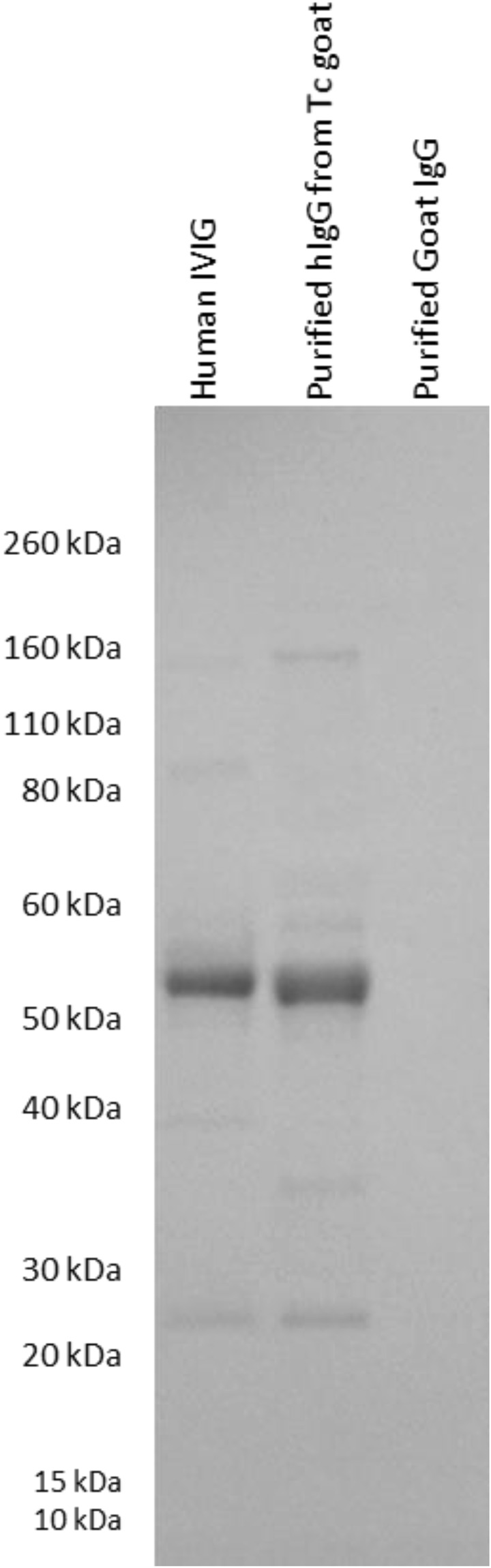
Confirmation of hIgG expression in the Tc goat with Western blotting. The purified hIgG from the Tc goat, positive control human IVIG, and negative control purified from commercial goat IgG was probed with anti-human IgG (heavy and light; H + L) HRP.

To evaluate human IgG levels in the Tc goat, human IgG ELISA was performed on the Tc goat serum samples collected at 3 weeks to 8 months of age. Three to 10 μg/ml of human IgG was detected in the Tc goat sera, which is similar to those observed in Tc bovine when the bovine Ig genes are intact at the similar developmental ages^3^. The production of hIgG in the Tc goat was also further confirmed by Western blotting experiments on purified IgG samples collected from its sera (Fig. 3).

### Characterization of antigen-specific hIgG produced in the Tc goat

**Figure 4:**
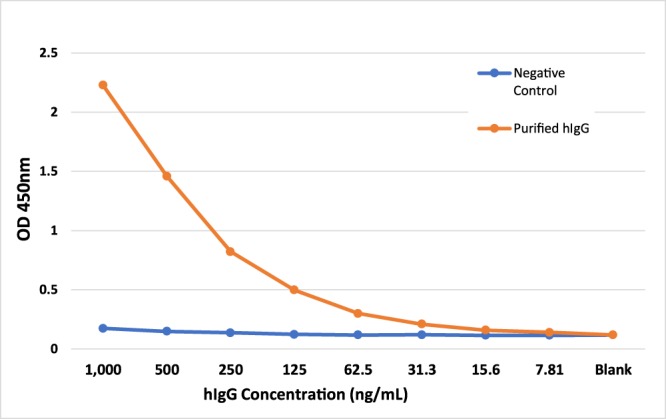
Characterization of antigen-specific hIgG produced in the Tc goat. H7N9 HA-specific hIgG titers in purified hIgG from the immunized Tc goat were compared to negative control hIgG purified from unimmunized Tc animal.

To investigate whether the Tc goat can mount hIgG-mediated humoral immune response to antigens, we immunized the Tc goat at the age of 5 months with inactivated H7N9 virus for the first and second rounds of vaccinations followed by recombinant H7N9 HA1 for the third and fourth vaccinations. At day 8 and 15 post the fourth vaccination, serum samples (40 ml per collection) were collected from the immunized goat from which hIgG was purified. Antigen-specific hIgG titer was analyzed by H7N9 HA1-specific hIgG ELISA. As shown in Fig. 4, while no H7N9 HA1–specific binding was detected in the negative control hIgG isolated from an unimmunized Tc animal, high titers of H7N9 HA1–specific hIgG were detected from the hIgG purified from the sera of the Tc goat post H7N9 HA1 immunization. We concluded that the HAC confers the Tc goat with the capability of generating antigen-specific hIgG response to antigen challenges.

We then conducted virus neutralization assays to investigate the potency of purified hIgG against H7N9 viruses. We demonstrated that hIgG purified from the H7N9 immunized Tc goat had high neutralization titer activity against H7N9 virus (Table 3). This indicates that hIgG elicited by the H7N9 immunization regimen in the Tc goat neutralizes live H7N9 viruses with high potency *in vitro*.

**Table 3:** H7N9 virus neutralization activities.

Sample	hIgG Concentration (mg/ml)	Neutralization Titer*	Neutralization Titer Activity (ug/ml)^
purified hIgG	0.75	200	3.75
Negative antibody Control	1	0	>1000

*Neutralization Titer is the dilution of the antibody solution that neutralizes H7N9 virus.

^^^Neutralization Titer Activity is the concentration of human IgG required to neutralizes H7N9 virus.

## Discussion

To address the challenge of insufficient supplies of hIgG, extensive research has been carried out to develop alternative strategies for the production of large quantities of hIgG. We recently reported our success in establishing a Tc bovine platform that allows, for the first time, the production of large quantities of highly potent hIgG (300–600 g of hIgG per animal per month)^6^. Because Tc animals are raised in biosecure facilities with animal pathogen control measures in place, the Tc platform eliminates disease transmission from human donors to patients, a common concern in using human IVIG products. Moreover, Tc animals can be hyperimmunized with a pathogen of choice formulated with proprietary adjuvants developed at SAB Biotherapeutics to produce high titers of pathogen-specific hIgG, overcoming the low potency problems associated with human IVIG produced from general human donor populations. Indeed, by using several emerging viral infection diseases as the targets, we have demonstrated in animal models that pathogen-specific hIgG produced from the Tc bovine system is highly potent in treating these diseases^6–9,11–15^, with some of them having already been in clinical trials^7,13^.

In this report, we describe our efforts to extend the Tc technology to a smaller ungulate, the goat (caprine). For certain biomedical applications, such as for serological testing and as therapeutics for small patient populations, it is more desirable to produce smaller volumes of hIgG products. It is also highly desirable to have the capability of swiftly establishing a Tc animal heard for therapeutic hIgG production in responding to sudden outbreaks of infectious diseases. In this regard, the goat offers the advantages of having a much shorter reproductive cycle and physical maturation time than larger ungulates. Furthermore, in addition to allowing plasma pooling strategies to be employed from a large number of Tc animals for greater lot to lot consistency, each of the Tc goats can be used for producing personalized-therapeutics at an affordable cost.

With the successful development of the first Tc goat and the production of pathogen-specific hIgG, we have demonstrated the feasibility of the Tc technology in the goat. However, this is only the first step in our efforts to fully establish the Tc goat system: because the goat endogenous Ig genes are still intact in the Tc caprine reported here, it is most likely that, as demonstrated in Tc bovine^3^, the endogenous goat Ig genes are predominantly expressed over the human Ig genes in goat B cells. The competition between goat Ig genes and human Ig genes would severely suppress the expression of human Ig genes leading to low level expressions of hIgG, a phenomenon that we have observed in the Tc bovine system when the bovine Ig genes are intact^3^; in addition, the expression of endogenous goat Ig genes may also lead to the formation of chimeric antibodies—in certain percentages of the produced antibodies, the light chains are of goat origin while the heavy chains are of human origin, or vice versa. To address these issues, we have initiated the efforts to genetically inactivate the goat Ig genes to greatly enhance the production of hIgG in the Tc goat system and to prevent chimeric antibody production, a strategy that has proven effective in Tc bovine^16^. We have recently knocked out the goat Ig heavy chain, lambda light chain, and kappa light chain genes (triple knockout) in the same goat primary fibroblast cells. These triple knockout cell lines are being used for Tc goat production. Our goal is to achieve a hIgG production level in the Tc goat system comparable to the goat IgG levels in wild type goats^17^, an objective that has been achieved in the Tc bovine system. To test if the Tc caprine is fully fertile, we recently bred it with a wild type ram, and as the time of submitting this report, the Tc caprine was detected pregnant (with two live fetuses) by ultrasound at a gestation stage of about day 50. As normal fertility of Tc goats is important for quickly expanding a Tc herd, the success of this Tc goat in establishing pregnancy is encouraging, even though we are still awaiting the outcome at full term of the pregnancy.

Tc goats can be hyperimmunized multiple times with vaccines containing strong adjuvants and/or immune stimulators, leading to the production of highly potent neutralizing hIgG against any pathogen of choice. Because a large herd of Tc goats can be produced quickly, the Tc caprine system can provide patients with hIgG products that are highly potent and safe at low cost. Our immunization studies with H7N9 followed by functional characterizations of the H7N9-specific hIgG elicited from the Tc caprine have demonstrated the proof of concept of this novel Tc caprine system for such therapeutic applications. It is also our intention that hIgG products produced from hyperimmunized Tc goats will be used as diagnostic reagents where human antibodies are desirable.

In summary, we have successfully extended our Tc technology to the goat leading to the production of a Tc goat that expresses hIgG in its sera. We also demonstrated that the Tc goat can be hyperimmunized with a pathogen of choice to elicit high titers of pathogen-specific hIgG (see Fig. 4 and Table 3). Therefore, the Tc goat system may provide a novel solution to the production of target-specific and high titer human antibodies for both therapeutic and diagnostic applications.

## Methods

All animal procedures were approved by and conducted according to the guidelines of the Utah State University Animal Care and Use Committee. All chemicals were purchased from Sigma–Aldrich (St. Louis, MO, USA) unless otherwise specified.

### Oocyte collection

Ovaries from domestic goats *(Capra aegagrus hircus*) were obtained from a local abattoir (Springville, UT, USA) and transported to the laboratory within 4 hours after collection. The ovaries were transported at 20–27 °C in saline containing 100 U/mL penicillin/ streptomycin. On arrival at the laboratory, the ovaries were processed using a slicing technique as previously described^18^. Only oocytes with three or more layers of compact cumulus cells and homogeneous cytoplasm were used.

### *In vitro* maturation

*In vitro* maturation was performed as previously published by our group^18^. Briefly, the cumulus oocyte complexes (COCs) were cultured in groups of 50 in 4-well plates containing 500 mL of maturation medium (TCM-199 [Gibco, Grand Island, NY, USA], containing 10% (vol/vol) fetal bovine serum (FBS), 10 μg/mL LH, 5 μg/mL FSH, 1 μg/mL estradiol-17β, and 0.05 g/L gentamicin. After 22 hours of culture, cumulus cells were removed from matured oocytes by vertexing the COCs for 1–2 min in TL-Hepes containing 1 mg/ml hyaluronidase. Nuclear maturation was confirmed by the presence of a first polar body. Oocytes at this stage are termed MII oocytes.

### HAC vector transfer into goat fibroblasts

Goat fibroblasts were cultured in α-MEM (HyClone) medium supplemented with 15% (vol/vol) FCS (HyClone, Logan, UT, USA) at 37 °C and 5% CO_2_. Microcells were purified from the CHO clone retaining the isKcHAC∆ as described previously^6^. Goat fibroblasts were fused with microcells using polyethylene glycol (PEG 1500), and the fused cells were selected under 600 µg/ml of Zeocin (Life Technologies, Carlsbad, CA, USA) for 14–21 days. The Zeocin-resistant clones were screened to confirm the presence of HAC in the cells and were subsequently used for SCNT.

### Somatic cell nuclear transfer

HAC-containing fetal fibroblast cells were cultured in DMEM/high-glucose medium (HyClone, Logan, UT, USA) supplemented with 15% (vol/vol) FBS and 100 U/mL penicillin/streptomycin. The fibroblasts were grown to 80 to 90% confluence and used as nuclear donor cells for SCNT after 24 to 48 hours of serum starvation (0.5% FBS). SCNT was performed as described before^18^. Briefly, a polar body and metaphase plate were removed from an MII oocyte and a single donor cell was subsequently transferred into the perivitelline space of the enucleated oocyte. Fusion was performed in the 0.28 M sorbitol fusion medium (0.1 mM calcium, 0.5 mM magnesium, 0.5 mM Hepes, and 1 g/mL BSA) by a single DC electric pulse of 1.75 kV/cm for 15 microseconds. Fusion of the donor cell with the oocyte cytoplasm was evaluated by microscopy 30 minutes after the pulse. After fusion, embryos were held in synthetic oviductal fluid (SOF) medium^19^. Fused embryos were activated between 27 and 29 hours after the onset of maturation by exposure to 5 μM ionomycin for 5 minutes followed by a 4-hour incubation in 2 mM DMAP and 10 μg/mL cycloheximide. Then embryos were cultured under oil in 50 μL droplets of SOF medium for 8 to 12 hours before the transfer into the estrus synchronized recipient females.

### Recipient synchronization and embryo transfer

Recipient synchronization and embryo transfers were conducted as described elsewhere^18^. Briefly, SYNCRITE Vaginal Sponges (Animal Health Supplies, Ulladulla, Australia) containing 40 mg of flurogesterone acetate were placed intravaginally for 10 days. Estrus occurred at 36 to 48 hours after sponge removal, and ovulation usually occurs at 12 to 24 hours after the occurrence of estrus. Fourteen domestic goats 2–5 years of age (*Capra aegagrus hircus*) were used as recipients for embryo transfers. Somatic cell nuclear transfer pregnancies were established by surgically transferring 14 ± 3 one-cell stage embryos into the oviduct of synchronized recipients that exhibited estrus within 12 hours of SCNT. Pregnancy confirmation was done 40 ± 3, 60 ± 3, and 90 ± 3 days after embryo transfer by transabdominal ultrasonography. All animals containing a viable conceptus as determined by the presence of a heartbeat were considered pregnant. After birth, the offspring were allowed to remain with the dams and nurse freely until weaning at 3 months of age.

### PCR analysis of HAC retention and integrity in Tc goat blood cells

DNA was extracted from goat whole blood collected at birth of Tc goat using the Gentra Puregene Kit (Qiagen 158422). These analyses were implemented as previously described^3,4^. All the PCR products were run on 0.8% agarose gels. Primer sequences are provided in Table 2.

### Human IgG ELISA

For detection of human IgG in Tc goat sera, human IgG ELISAs were performed in Maxisorp Immuno 96-well ELISA plates as described previously^6^. Briefly, goat anti-human IgG-Fc (Bethyl, Montgomery, TX, USA) was used as a capture antibody. Goat anti-human IgG-Fc Antibody HRP conjugated (Bethyl, Montgomery, TX, USA) was used as a detection antibody. Human Reference Serum with known human IgG concentration (Bethyl, Montgomery, TX, USA) was used as standard. Wild type goat serum was used as a negative control.

### Inactivated H7N9 virus antigen preparation

Influenza A/Anhui/1/2013 H7N9 was grown in 9-day embryonated eggs and incubated at 37 °C for 72 hours. The allantoic fluid was harvested and titrated by plaque assay and ICID_50_. The virus was then inactivated by β-proprolactone (BPL) for 72 hours at 37 °C. The inactivation was tested by HA and then was injected into three 9-day embryonated eggs (100 μl/egg) and incubated at 3 °C for 72 hours before going to 4 °C for 24 hours. The allantoic fluid was then tested by HA and 100 μl of pooled negative and positive samples were inserted into three 9-day embryonated eggs for each. After incubating at 37 °C for 72 hours, the eggs were then put at 4 °C for 24 hours. The allantoic fluid was then tested for HA and BPL was repeated if necessary.

### Recombinant H7N9 HA1 antigen preparation

The H7N9 HA1 sequence was synthesized by genscript and delivered in the pUC57 vector. It was amplified using 15RI-HA1-F (TCTTGCACTTGTCACGAATTCGGATAAGATTTGTCTGGGCCATCATGCTGTGA) and 15SA-HA1-R (TGTGTGAGTTTTGTCTCTCCCCTTTGGAATCTCGGGCACAT) to generate the fragment for the human Fc (hFc) vector (pFUSEss-CHIg-hG1 expression vector (InvivoGen, Inc.) and 15RV-HA1-F (CTTGTCACGAATTCGGATAAGATTTGTCTGGGCCATCATGCTGTGA) and 15Bg-HA1-R (GCACGTGGGCTTGCTTCTCCCCTTTGGAATCTCGGGCACAT) for the rabbit Fc (rFc) expression vector, pFUSE-rIgG-Fc2 (IL2ss) (InvivoGen, Inc. pfuse-rfc2). PCR cloning was conducted using the CLONEZ system (Genescript L00339) and Subcloning Efficiency™ DH5a™ Competent Cells (Invitrogen 18265-017) for both vectors.

Transient expression of the recombinant H7N9 HA1-hFc or H7N9 HA1-rFc was accomplished using the Expi293F™ Cells (Life Technologies A14527) and the ExpiFectamine™ 293 Transfection Kit (Life Technologies A14524) following their standard protocol. After 6 days the supernatant was centrifuged at 3,200 rpm for 5 min and filtered (EMD Millipore S2GPU05RE) prior to purification. The recombinant H7N9 HA1-hFc or H7N9 HA1-rFc was purified through protein A column. The recombinant H7N9 HA1-hFc was use as the antigen for Tc goat vaccinations. The recombinant H7N9 HA1-rFc was used as coating antigen for H7N9 HA1-specific human IgG ELISA.

### Tc goat H7N9 immunization

Tc goat was immunized four times with 3-week intervals. The BPL-inactivated H7N9 virus (Influenza A/Anhui/1/2013 H7N9) formulated with SAB-adj-2 was given to Tc goat via intradermal (i/d) injection for the first vaccination at 0.25 mg per dose and the second vaccination at 0.5 mg per dose. Then the recombinant H7N9 HA1-hFc (0.6 mg HA1 per dose) formulated with SAB-adj-1 was given to Tc goat via intramuscular injection for the third and fourth vaccinations. Forty ml of serum per collection was collected from immunized Tc goat on day 8 and day 15 after the fourth vaccination for antibody purification.

### Purification of human IgG from Tc goat sera

Human IgG from Tc goat sera was first affinity-purified with an anti-human IgG Fc–specific column, IgSelect (GE Healthcare Life Sciences, Marlborough, MA, USA). Then human IgG was further purified by passage over an anti-goat IgG Fc–specific affinity column to remove remaining goat IgG. The purified human IgG has a IgG concentration of 0.75 mg/ml in a sterile-filtered PBS buffer.

### Western blotting

Immunoglobulin heavy and light chains of purified human IgG and goat IgG were separated by SDS PAGE using 4–12% precast Bis-Tris gels (Invitrogen) and transferred to polyvinylidene difluoride membranes that were directly probed with donkey anti-hIgG (H + L) specific horseradish peroxidase (HRP)-conjugated antibody (Jackson ImmunoResearch) for hIgG heavy chain and light chain detection.

### H7N9 HA-specific human IgG ELISA

To determine H7N9 HA-specific hIgG titers, 96-well Nunc Maxisorp ELISA plates were coated overnight at 4 °C with 100 μl/well of 2 μg/mL recombinant H7N9 HA1 (H7N9 HA1-rFc) in PBS. Plates were washed with PBST (PBS with 0.05% Tween 20) and blocked at room temperature (RT) for 1 hour with 1% casein (Thermo) in PBS. After washing with PBST, purified human IgG from immunized Tc goat and the negative control human IgG purified from unimmunized Tc animal were added to the plates with serial dilution in PBST and incubated for 1 hr at RT. Following washing with PBST, goat anti-human IgG-Fc conjugated with horseradish peroxidase (HRP) (Bethyl) was added to plates and incubated for 1 hr at RT. After final washing with PBST, the bound anti-H7N9 HA antibodies were detected colorimetrically by using a TMB substrate kit. The absorbance was read in a microplate reader at 450 nm.

### H7N9 neutralization test

MDCK cells were maintained in minimum essential medium (MEM) supplemented with 5% (vol/vol) FBS at 37 °C. Serially diluted purified human IgG and negative control in serum-free MEM with TPCK-treated trypsin (Sigma) was mixed with an equal volume of virus (1 × 10^5^ PFU/ml) and incubated for 1 h at 37 °C. Confluent MDCK cells in 96-well format were washed twice with PBS, the mixture antibody/virus was added to the cells and incubated for 72 h at 37 °C under 5% CO_2_. After the antibody/virus mixture was removed, fixed plates and stained with crystal violet for 30 minutes at RT for determining viability of cultured cells.
